# Editorial: Environment and organization sustainability: an employee perspective

**DOI:** 10.3389/fpsyg.2023.1241653

**Published:** 2023-07-28

**Authors:** Sikandar Ali Qalati, Mingyue Fan, Jian Zhou, Mónica Lorena Sánchez Limón, Xiaobo Chen

**Affiliations:** ^1^School of Business, Liaocheng University Liaocheng, Shandong, China; ^2^School of Finance and Economics, Jiangsu University, Zhenjiang, China; ^3^School of Business, Nankai University, Tianjin, China; ^4^Faculty of Commerce and Administration Victoria, Autonomous University of Tamaulipas, Ciudad Victoria, Mexico; ^5^School of Computer Science and Technology, Shandong Institute of Business and Technology, Yantai, China

**Keywords:** employee engagement, employee green behavior, employee green consumption, environmental sustainability, employee environmental performance

Currently, a topic of great importance is the sustainability of organizations and how it influences the care of the environment, since as we know we are going through moments of great environmental concern such as global warming and water scarcity, to name a few. This has caused great concern for the environment and the decrease in the availability of resources, since it is undoubtedly a subject of great impact, both personally and organizationally, for this reason it is important to know the factors that affect the of green behaviors and the importance of their influence on the economy of organizations, a key factor in this are the employees, since they are an important and fundamental part of spending many hours in the workplace, so thinking about them and take them into account to positively influence the environment, organizations, and personal life.

Global demand for resources continues to increase due to population growth and urbanization, causing resource depletion and a challenging environmental sustainability worldwide (Jiang and Wu).

The term “sustainability” refers to a company's ability to maintain a balance between profits, the environment and people. Most of the world's governments have established administrative law frameworks that bind public, private and multinational companies to comply with established environmental management standards and procedures. Researchers have examined the three pillars of sustainability: social, economic, and environmental (Lashari et al.).

Organizations should foster responsible leadership, which is defined as an ethical and moral approach to managing an organization that takes into account the needs and expectations of all interested parties, including employees, customers, suppliers, investors, and the community. Responsible leadership and management of all stakeholders can have a positive effect on an organization's bottom line, including improving its reputation, reducing risk and expense, increasing employee satisfaction and engagement. Similarly, responsible leadership and stakeholder management can influence innovation and creativity in organizations (He et al.; Zhou et al.).

According to reports, multinational companies have started to integrate sustainability into their management styles, while small and medium-sized enterprises (SMEs) are less committed. According to a study carried out by Galván-Mendoza et al., women show more green behaviors than men. This implies any conduct that promotes the preservation of natural resources, respect for the environment and any actions that support ecosystems balance. Green behavior has the effect of reducing negative results of daily activities at work because each employee's behavior contributes to achieving organization's sustainability (Jiang and Wu).

Sustainability can be examined form various angles. Lashari et al. argue that human resource management practices can help organizations meet the expectations of those interested in environmental sustainability. For example, they can help companies reduce their ecological footprint and improve their energy efficiency.

According to Zhou et al. employees are a key resource for an organization's environmental practices, and their behavior is an essential factor in achieving the company's sustainability objectives. Employees' respectful behavior toward the environment will define the positive or negative degree toward their working environment, which can reduce costs within the organization and directly benefit the company, as well as its efforts to protect the environment and natural resources as a whole. Hence, showing corporate responsibility and achieving sustainability.

The study by Costa et al. focuses on employees' pro-environmental behavior. In addition, the importance of a green organizational environment and its effect on employees is discussed. Various theoretical perspectives on sustainability analysis are mentioned, including the self-determination theory and the organizational support perspective.

Yan et al. provide empirical evidence that demonstrates a positive relationship between corporate social responsibility and financial results. Therefore, the importance of technological innovation and ecological growth in organizations should be an incentive to generate cooperation and economic development.

Galván-Mendoza et al. discuss the importance of employees in the sustainability and they prove that creating a propitious work environment, where employees feel supported by their organization, increases the employees' willingness to get involved in behaviors that protect nature.

In conclusion, sustainability is emerging as a potential competitive advantage for many existing organizations. In addition, it underlines how crucial it is to incorporate sustainability into the organizational theory and employee behavior within the company. Said integration is achieved through a channel among companies in the sector and their community, where they can respond to environmental concerns with their own internal ecological efforts. Likewise, govern and academia encourage companies to adopt strategies based on environmental performance and on their research.

Finally, it is important to highlight that leaders have the duty to act as information and knowledge intermediaries, capable of acquiring and disseminating relevant data associated with the organization's sustainability, as well as the preservation and protection of the environment. This knowledge sharing process enables employees to acquire the environmental skills and abilities they need to improve their own performance in the environment. Responsible leaders also value training and employees' empowerment in environmental protection and sustainable development, rewarding “environmental pioneers” and helping them understand broader corporate social responsibility. Spreading an overall message promoting sustainability and awareness is also part of ecological consumption through public advertising.

## Author contributions

SQ, MF, JZ, MS, and XC contributed to the conception and design of the study. All authors contributed to the revision of the manuscript, read and approved the submitted version.

